# Impact of *Bacillus coagulans* Fortification on Storage-Induced Metabolomic Changes in Extruded Mung Bean Snacks

**DOI:** 10.3390/biology15141113

**Published:** 2026-07-09

**Authors:** Jutamat Klinsoda, Theera Thurakit, Orawan La-Ongkham, Pramuan Saithong, Napassorn Peasura, Wanida Pan-Utai, Khemmapas Treesuwan, Kanokwan Yodin, Hataichanok Kantrong, Jirawut Permpool, Kanthida Wadeesirisak

**Affiliations:** Institute of Food Research and Product Development, University of Kasetsart, Bangkok 10903, Thailand; ifrtet@ku.ac.th (T.T.); ifrowl@ku.ac.th (O.L.-O.); ifrpmst@ku.ac.th (P.S.); ifrnop@ku.ac.th (N.P.); ifrwdp@ku.ac.th (W.P.-U.); khemmapas.tr@ku.th (K.T.); ifrkwd@ku.ac.th (K.Y.); ifrhnk@ku.ac.th (H.K.); jirawut.6106@gmail.com (J.P.)

**Keywords:** metabolome, probiotic, extrusion, mung bean

## Abstract

**Background:** Incorporating probiotic bacteria such as BC, a spore-forming probiotic bacterium known for its resistance, into extruded snacks has emerged as an innovative approach to developing functional foods. Little is known about how the addition influences metabolomic changes during storage. **Methods:** The mung bean snacks were produced by extrusion, followed by BC fortification. The control snack without seasoning and the two BC treatments (0.1% and 0.5% *w*/*w*) were stored at room temperature for 3 months. Culture-based methods and qPCR were performed for microbial analysis. Metabolites were extracted, and metabolite profiles were evaluated using an LC-MS-based approach. **Results:** 0.5% BC was the most stable in the snack, with viable counts of 7.10 log CFU/g. The PCA plot demonstrated distinct clustering patterns among groups, despite no significant difference by PERMANOVA. In negative mode, L-tyrosine showed higher accumulation in the 0.1 BC group, whereas Threonine, L-histidine, and fatty acid metabolites were highly abundant in the 0.5 BC treatment. The positive mode showed discriminant metabolites, with L-glutamic acid, Isoleucine, and L-phenylalanine enriched in the 0.5 BC treatment. Overall, BC altered citrate and phenylalanine metabolism, and each treatment affected aminoacyl-tRNA biosynthesis. **Conclusions:** These findings demonstrate the maintenance of culturable *B. coagulans* in fortified extruded mung bean snacks during storage and provide exploratory insights into metabolite changes associated with amino acid metabolism, lipid oxidation, and flavor development that may be relevant to product quality and storage stability.

## Simple Summary

Fortification of probiotics in snack products remains challenging due to the need to ensure their survival. In the present study, different levels of the spore-forming probiotic *Bacillus coagulans* (BC) were coated on the extruded mung bean snacks. An omics approach (metabolomics) was used to evaluate the influence of *Bacillus coagulans* fortification on metabolite dynamics and storage stability of extruded mung bean snacks. Microbial analysis revealed that BC increased in the snacks during storage, while metabolites reflected a shift in bacterial metabolism. These findings provide new insights into the complex dynamics of metabolites associated with BC fortification, which provide a critical foundation for optimizing the shelf life of probiotic-fortified extruded mung bean snacks.

## 1. Introduction

Increasing awareness of health and sustainability issues has accelerated the development of plant-based functional products. Legumes have attracted considerable attention for their high protein content, dietary fiber, and diverse phytochemicals. The global Mung Bean Market is on a steady upward trajectory, projected to grow from USD 4.63 billion in 2025 to USD 5.79 billion by 2033 at a Compound Annual Growth Rate (CAGR) of 2.7% [1]. Mung beans (*Vigna radiata* L.) have gained interest as an important ingredient in dietary options. Mung beans contain approximately 22–28% protein, along with amino acids, phenolic compounds, flavonoids, and other antioxidants, which provide several health-promoting benefits [2]. Their growing role as a foundational ingredient in snacks, beverages, and processed foods further amplifies their commercial and industrial value [1]. Mung bean-based flour is suitable for incorporation into value-added snack products to increase nutritional and functional value. In the food industry, extrusion is widely used to convert legume flours (e.g., soybean flour and mung bean flour) into ready-to-eat foods, snacks, and protein products. Extrusion technology uses a high-temperature, short-time (HTST) processing technique, which promotes starch gelatinization, enhances protein digestibility, and reduces antinutritional compounds such as phytic acid and tannins, creating a desirable texture and extending the product’s shelf life [2,3,4].

Currently, the growing popularity of plant-based foods and interest in probiotic-enriched foods is driven by consumers becoming more aware of the relationship between diet and gut health. Probiotics are live microorganisms that provide health benefits to the host when consumed in sufficient amounts. Probiotics are involved in supporting digestive health, regulating immune responses, and maintaining a healthy balance of intestinal microbiota [5,6]. Despite their benefits, incorporating conventional probiotic bacteria such as *Lactobacillus* and *Bifidobacterium* into thermally processed foods is difficult. These microorganisms are sensitive to heat, oxygen, and dehydration, which can reduce their viability during processing. In extrusion-based products, they are often inactivated by the high temperature and pressure inherent to the extrusion process, as high temperatures and pressures involved often result in substantial losses of probiotic cells [6].

Spore-forming probiotic bacteria have emerged as promising alternatives due to their enhanced environmental resistance. *Bacillus coagulans* has become an innovative candidate and a focus of commercial interest because of its ability to form highly resistant endospores [7,8]. It exhibits exceptional resilience to thermal processing and tolerates extreme environmental conditions, including high temperatures, desiccation, and acidic environments, while maintaining stability during gastrointestinal transit [9]. *B. coagulans* forms spores, which can survive harsh food processing conditions and maintain viability during storage. Several studies have reported its stability in a range of food products, such as cereals, baked products, and dietary supplements [7,8]. In addition, clinical and experimental studies suggest that the consumption of *B. coagulans* may contribute to improved digestive function [9,10], beneficial changes in gut microbial composition, and enhanced immune responses [11,12]. With these characteristics, *B. coagulans* becomes an attractive probiotic candidate for incorporation into food products, particularly those produced under intensive processing conditions such as extrusion [10]. While its survival is well documented, the storage-induced metabolic dynamics within the food matrix—and the extent to which the presence of *B. coagulans* shapes these complex biochemical changes over time—remain poorly understood in food science.

To investigate biochemical changes, metabolomics, an advanced high-throughput omics approach, offers a powerful platform generating a detailed metabolic fingerprint of a sample [13]. This technique enables the examination of biochemical transformations occurring within food systems driven by enzymatic activity and oxidative reactions. This approach can also be used to identify metabolites and key biomarkers. The assessment of metabolic activity can identify quality deterioration of food matrices during storage [14]. Previous studies have shown that metabolomics is a useful tool for understanding biochemical changes during food storage. For instance, an untargeted UHPLC-MS/MS metabolomics study on refrigerated beef found that the interaction between *Pseudomonas lundensis* and *Brochothrix thermosphacta* changed histidine metabolism, with creatine and inosine identified as important spoilage biomarkers [15]. Another study on the metabolome of soy sauce products has revealed that Maillard reactions and carbohydrate degradation during storage contribute to the formation of compounds linked to undesirable bitter and sour tastes [16]. Metabolomics has been used to study several milk products incorporated with probiotics. Recent research using LC-MS/MS-based metabolomics and peptidomics identified 147 peptides and 39 peptides in fresh cheese fermented with three novel probiotics (*Lacticaseibacillus rhamnosus* B6, *Limosylactobacillus fermentum* B44, and *Lacticaseibacillus rhamnosus* KF7), confirming that the presence of probiotics directly accelerates the release of functional peptides [17]. In plant-based food, Shudong et al. [18] revealed that the 17 major metabolic changes in a probiotic beverage from soy protein and fructooligosaccharide fermented by *B. coagulans* VHProbi C08, but the properties of the plant-based drink were relatively stable during 28 days of storage [18].

Although *B. coagulans* probiotic-fortified snacks have received increasing research attention, previous research has focused on probiotic viability and consumer acceptance. The metabolome dynamics of these products during storage remain poorly understood, particularly in plant-based snack systems. Uncovering the interactions between *B. coagulans* and the mung bean snack may provide valuable insights into product stability, safety, and functional performance. *B. coagulans* has received increasing attention for application in processed and shelf-stable foods because of its spore-forming ability. Technologically, this spore-forming property is advantageous for food matrices exposed to processing and storage stresses, as it can improve probiotic survival during product handling and ambient storage. In cereal-based extruded snacks, however, extrusion involves high temperature and mechanical shear, which may still reduce probiotic viability if cells or spores are incorporated directly into the dough. In the present study, thus, *B. coagulans* SNZ1969 was applied as a post-extrusion probiotic seasoning after cooling of the snack base, using the spore-forming nature of the organism together with post-extrusion incorporation and high-barrier nitrogen-flushed packaging as a strategy to preserve probiotic viability during storage. The present study aims to investigate the storage-driven dynamics of the metabolome in *B. coagulans*-fortified extruded mung bean snacks using untargeted metabolomics and pathway enrichment analysis. The findings will provide new insights into the biochemical mechanisms underlying product stability and help identify potential metabolites associated with metabolomic changes during storage. Ultimately, this study may contribute to the development of reliable shelf-life indicators for the next generation of probiotic-fortified extruded foods.

## 2. Materials and Methods

### 2.1. Raw Materials and Sample Preparation

Mung bean (*Vigna radiata* L.) flour, cereal flour (rice and corn), and soy flour were used as the primary ingredients for the extruded snack formulation. Hulled mung bean flour was prepared through cleaning and milling to obtain finely ground mung bean flour. The snack base was formulated using mung bean flour, cereal flour, and soy flour at a ratio of 60:30:10 (*w*/*w*), as adapted from the optimized mung bean snack formulation reported by Boonyasirikul et al. [19]. In the present study, the formulation was modified for probiotic snack development by applying *B. coagulans* SNZ1969 as a post-extrusion probiotic seasoning rather than incorporating the probiotic into the dough before extrusion. Commercial seasoning powder and powdered *B. coagulans* SNZ1969 were used for the preparation of probiotic seasoning. A commercial dried probiotic preparation of *B. coagulans* SNZ1969 15 MLD (lot C397058A; Sacco, Como, Italy) was used as the probiotic ingredient in this study. The preparation was used as received from the supplier and was not propagated, harvested, or further processed in the laboratory before incorporation. According to the supplier’s certificate of analysis, the preparation contained 2.3 × 10^10^ CFU/g and complied with the specified microbiological purity criteria for *Enterobacteriaceae*, *Escherichia coli*, foreign yeasts, molds, non-lactic acid bacteria, coagulase-positive *Staphylococci*, *Salmonella*, *Listeria monocytogenes*, and *Bacillus cereus.* The water activity of the commercial powder was 0.305.

### 2.2. Extrusion Processing Conditions

Extrusion was performed using a co-rotating twin-screw extruder (model ZE25x33D, Hermann Berstorff Laboratory, Hanover, Germany) with L/D 870:25. The extrusion conditions were adapted from Boonyasirikul et al. [19] and Charunuch et al. [20], who reported optimized processing conditions for mung bean-based extruded snacks produced using a twin-screw extruder. The feed moisture content was adjusted to 16%, and the screw speed was set at 300 rpm. The highest barrel temperature was controlled at approximately 135 °C, with the overall processing temperature maintained within the range of 90–160 °C. The feed rate was 16 kg/h, and the product was extruded through a 3.0 mm die to obtain expanded snack products. The extrusion process was conducted at a specific mechanical energy (SME) of approximately 80 Wh/kg (≈288 kJ/kg), reflecting a moderate-to-high thermo-mechanical energy input. SME is widely recognized as an integrated indicator of mechanical shear and viscous dissipation during extrusion and is closely associated with the extent of structural transformation of starch- and protein-based matrices. After extrusion, the snacks were cooled to room temperature before seasoning application. No *B. coagulans* SNZ1969 was present in the dough or snack base during extrusion. The extrusion step was used only to produce the unseasoned expanded snack base; therefore, the probiotic spores were not exposed to extrusion-related thermal shock, high temperature, or mechanical shear.

### 2.3. Preparation of Probiotic Seasoning and Snack Coating

The probiotic seasoning was prepared by mixing commercial seasoning powder with powdered *B. coagulans* SNZ1969. To promote homogeneous spore distribution, powdered *B. coagulans* SNZ1969 was first premixed with the commercial seasoning powder before application to the snack base. The probiotic-containing seasoning was then applied to the cooled extruded snacks using a stainless-steel rotating cubic seasoning mixer in the factory production line. During coating, the mixer rotated continuously, allowing the snacks and seasoning powder to tumble and contact repeatedly until the seasoning was visually and uniformly distributed over the snack surface. Because *B. coagulans* was incorporated by post-extrusion seasoning, spore distribution was controlled as a surface-coating process rather than by incorporation into the internal snack matrix. Since the probiotic was incorporated as a finished commercial preparation, no culture medium was used in the present experimental process, and no washing, centrifugation, or additional purification step was performed by the authors before incorporation. The seasoning powder was applied at 11% (*w*/*w*) based on the weight of the unseasoned extruded snacks. The amount of *B. coagulans* SNZ1969 powder was calculated as 0.1% (*w*/*w*) and 0.5% (*w*/*w*) of the total sample weight, including the unseasoned snack and seasoning powder, to obtain a probiotic level of at least 10^6^ CFU/g sample. After extrusion and cooling to ambient temperature (30 ± 2 °C), the snack samples were coated with the probiotic-containing seasoning powder under hygienic conditions. The probiotic was applied after extrusion to avoid direct exposure of *B. coagulans* SNZ1969 to the high temperature and mechanical shear associated with extrusion processing. The seasoned snacks were mixed until the seasoning powder was evenly distributed over the snack surface. The coated snacks were then immediately subjected to nitrogen-flushed packaging.

### 2.4. Storage Stability Study

The probiotic-seasoned snacks were packed using an automatic nitrogen-flushing packaging machine. An unprinted high-barrier aluminum-laminated film composed of PET/ALU/LLDPE (12/7/65 µm; total thickness 84 ± 5 µm) was used as the packaging material. The packages were heat-sealed immediately after nitrogen flushing to limit oxygen exposure and moisture uptake during storage and stored under ambient room-temperature (30 ± 2 °C) conditions for 12 weeks to evaluate real-time storage stability. Sampling was conducted at day 0 and 3 months for microbial analysis and metabolic profiling in 2 replicates (*n* = 2 bags/formulation).

### 2.5. Microbial Analysis

#### 2.5.1. Enumeration of Viable *Bacillus coagulans* During Storage

The viability of *B. coagulans* in the probiotic-seasoned snacks was determined at day 0 and after 3 months of storage using a heat-treatment spread-plate method adapted for the enumeration of aerobic spore-forming bacteria [21,22]. Briefly, 50 g of sample was aseptically transferred into a sterile stomacher bag and homogenized with 450 mL of sterile Butterfield’s phosphate-buffered diluent (BBP, pH 7.2) [23] using a stomacher (BagMixer 400, Île-de-France, France) at speed setting 4 for 1 min. The homogenate was then heat-treated in a water bath at 80 °C for 30 min to thermally inactivate vegetative cells and select heat-resistant spores [21]. After heat treatment, the suspension was centrifuged at 1000 rpm for 10 min, and the supernatant was serially diluted in sterile BBP to obtain decimal dilutions from 10^−1^ to 10^−6^.

A 0.1 mL aliquot from appropriate dilutions was spread-plated onto tryptone–yeast extract–glucose agar (TYGA, pH 5.5). TYGA was prepared using tryptone 5.0 g/L, yeast extract 3.0 g/L, glucose 1.0 g/L, and agar 15.0 g/L, with the pH adjusted to 5.5 before sterilization. The plates were incubated aerobically at 50 °C for 48 h. Typical colonies were counted, and the results were expressed as log_10_ CFU/g of sample. Plates within the countable range, preferably 25–250 colonies, were used for calculation according to standard aerobic plate count principles [22]. The counts were calculated using the following equation:

CFU/g = N/(V*D)

where N is the mean number of colonies, V is the plated volume in mL, and D is the decimal dilution of the plated sample relative to the original sample.

Unless otherwise confirmed by strain-specific identification, the results were reported as presumptive viable *B. coagulans* spores because the method quantified heat-resistant, aerobic spore-forming colonies under the specified selective conditions [24].

#### 2.5.2. DNA Extraction and Quality Control

Total genomic DNA was extracted from the snack samples (*n* = 2/day/formulation) using the Qiagen DNeasy^®^ mericon^®^ Food kit (Qiagen, Hilden, Germany), with minor adaptation of glass bead beating (0.5 mm) using MP FastPrep at 6.5 m/s for 3 × 30 s and then following the manufacturer’s protocols. The DNA was measured using a NanoDrop (Thermo Fisher Scientific Inc., Waltham, MA, USA) to ensure the purity (A260/A280 ratios of 1.8–2.0) before sequencing.

#### 2.5.3. Quantitative PCR (qPCR) Analysis

Specific primers for the total bacteria and *B. coagulans* were used (Table 1). Quantitative PCR was carried out using the SYBR Green qPCR Master Mix (No ROX) (MedChemExpress, Monmouth Junction, NJ, USA). The qPCR was performed in a total reaction volume of 20 μL containing 10 μL of Master Mix, 0.4 μL each of forward and reverse primers (10 μM initial concentration, 0.2 μM final concentration), 2 μL of DNA template, and 7.2 μL of nuclease-free water (ddH_2_O). Thermocycling was initiated with an initial denaturation at 95 °C for 5 min, followed by 40 cycles of denaturation at 95 °C for 10 s, annealing at 63 °C for 20 s, and extension at 72 °C for 20 s. To confirm amplification specificity and ensure the absence of primer dimers, a melt curve analysis was conducted immediately after completion of the amplification cycles, using a profile of 65 °C for 5 s and a final ramp-up to 95 °C for 50 s. All reactions were carried out using a CFX96™ System (Bio-Rad Laboratories, Inc., Hercules, CA, USA), and threshold levels were calculated using the 2^−ΔΔCT^ method compared to the control group of total bacteria in each treatment. DNA was used as the template, and each reaction, including a negative control, was conducted in triplicate.

### 2.6. Metabolome Analysis

#### 2.6.1. Sample Preparation and Extraction

To capture the dynamics of the food metabolome during storage, metabolic profiling was conducted on the extruded snacks (*n* = 2/day/formulation). The extract samples were resuspended in 250 µL of 0.1% formic acid prepared with LC-MS grade deionized water (CHROMASOLV™, Honeywell, Seelze, Germany). The resulting supernatant was then filtered through a 0.45 µm pore size hydrophilic nylon syringe filter. For analysis, 100 µL of the prepared sample was collected in glass vials with inserts in 3 replicates/day/formulation.

#### 2.6.2. LC-MS/MS Instrumentation and Conditions

Chromatographic separation was performed using a Dionex Ultimate 3000 HPLC system (Dionex, Thermo Fisher Scientific, Germering, Germany). An Acclaim Polar Advantage II C18 column (2.1 × 100 mm, 3 µm) was utilized, equipped with a corresponding guard column (3 × 10 mm, 5 µm). The injection volume was set to 3 µL, with the column and autosampler temperatures maintained at 40 °C and 10 °C, respectively.

The mobile phases consisted of (A) water with 0.1% formic acid and (B) acetonitrile with 0.1% formic acid. The separation followed a precise gradient program over a 30 min run. Detection was carried out using a Bruker compact QTOF mass spectrometer (Bruker Daltonics GmbH, Bremen, Germany). To identify active ingredients, MS signals were acquired in the *m*/*z* range of 50–1000 separately under both positive-ion and negative-ion electrospray ionization (ESI) modes. The ESI settings included a nebulizing gas pressure of 2 bars, a drying gas flow of 8 L/min, and a drying gas temperature of 220 °C. Three replicates per sample were performed.

#### 2.6.3. Data Processing and Metabolite Identification

The raw MS data were processed using MetaboScape^®^ 2022 software (Bruker Daltonics GmbH, Bremen, Germany) in the T-ReX 3D workflow. Identification of metabolites was based on mapping the MS/MS spectra and retention time in the Bruker MetaboBASE Personal Library 2.0 (Bruker Daltonics GmbH, Bremen, Germany).

### 2.7. Statistical Analysis

LC/MS-derived metabolite data were analyzed using multivariate statistical approaches in MetaboAnalyst 6.0. Principal component analysis (PCA) was applied to assess differences in metabolite profiles among experimental groups.

Random Forest analysis in MetaboAnalyst 6.0 was performed to identify metabolites contributing to group discrimination, and exploratory feature-ranking was performed according to Mean Decrease Accuracy values. The top discriminative metabolites were visualized using a heatmap of relative metabolite abundance.

In addition, pathway analysis was performed to identify altered metabolic pathways based on the Kyoto Encyclopedia of Genes and Genomes (KEGG) database. Metabolite data were normalized before statistical analysis. Differential metabolites were identified using a two-way mixed-effects ANOVA with treatment and storage time as fixed effects and biological replicate as a random effect. Multiple comparisons were adjusted using the Benjamini–Hochberg false discovery rate (FDR) procedure, and metabolites with FDR-adjusted *p*-values < 0.05 were considered significant. Permutational multivariate analysis of variance (PERMANOVA; 999 permutations) was performed to assess differences in overall metabolite profiles among groups. Graphical visualization and additional statistical analyses were conducted using GraphPad Prism 11.0.

## 3. Results

### 3.1. Characterization of B. coagulans in Fortified Extruded Mung Bean Snacks for 3 Months of Storage

Viable cell counts were determined using the plate count method on TYGA agar. The viable counts of *B. coagulans* with the initial count at 8 log CFU/g remained relatively stable throughout storage, indicating good survival of the probiotic spores in the extruded snack matrix. At the end of the three-month storage period, the viable count was 6.55 log CFU/g for the 0.1 BC formulation and 7.10 log CFU/g for the 0.5 BC formulation. The retention of viable counts above the recommended minimum level of 6–7 log CFU/g indicates that the product has the potential to deliver an effective probiotic dose throughout its intended shelf life.

**Figure 1:**
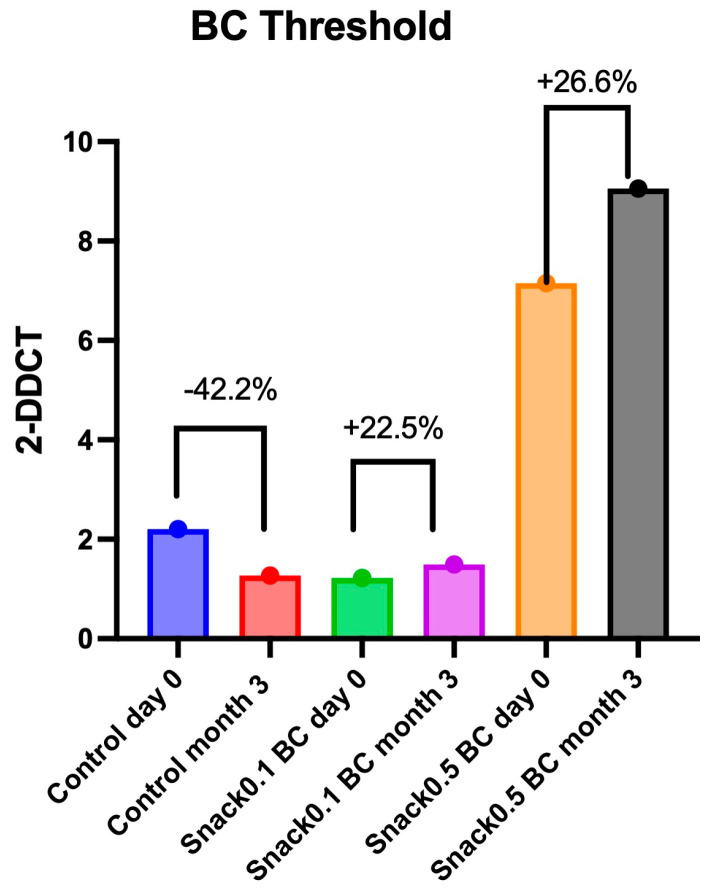
*Bacillus coagulans* threshold compared with the total bacteria by qPCR.

From the result of DNA abundance by qPCR, the addition of *B. coagulans* in extruded mung bean snacks resulted in a different *B. coagulans* threshold based on the treatments (Figure 1). Although without fortification of *B. coagulans*, the snack based on the control snack contained *B. coagulans* and was reduced by 42.2% within 3 months of storage. Both BC groups showed an increase in *B. coagulans* threshold during storage. The coating with 0.5%BC was considerably higher in *B. coagulans* threshold than 0.1% BC, and the % threshold increased by about 26.6%.

### 3.2. Metabolome

#### 3.2.1. Principal Component Analysis (PCA) from Negative Mode

**Figure 2:**
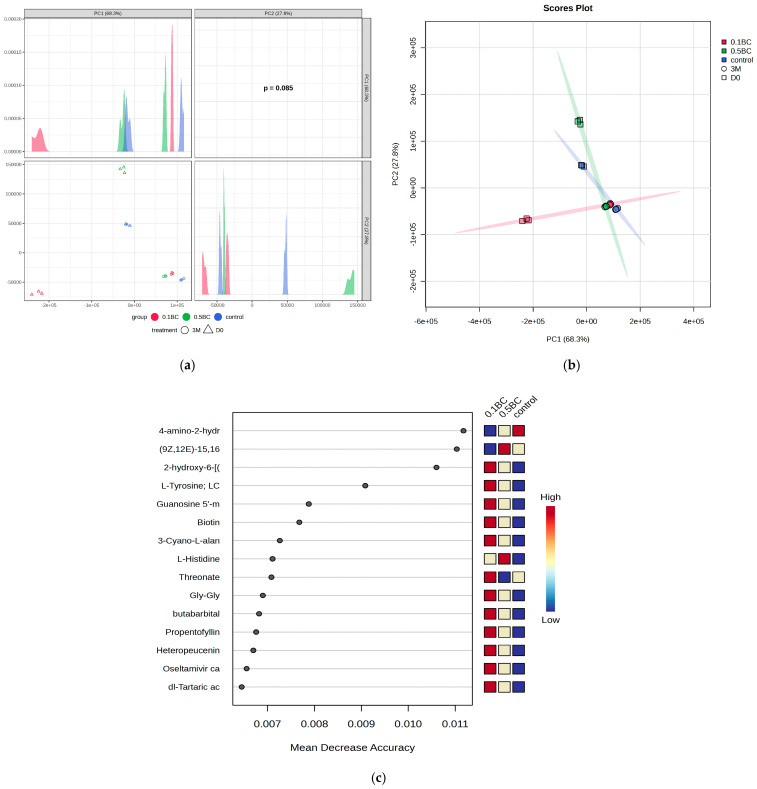
Principal components analysis (PCA) of Metabolite Profiles in the Snacks analyzed by GC-MS in Negative mode, (**a**) PCA, (**b**) PCA score plot, (**c**) Random Forest variable importance plot. The heatmap shows the relative abundance patterns across groups, with red indicating higher abundance and blue indicating lower abundance.

Principal component analysis (PCA) revealed partial separation among the experimental groups based on metabolite profiles (Figure 2a). The PCA indicated the metabolite profiles based on the treatment groups and the storage time, and clustering analysis suggested a tendency toward group discrimination (*p* = 0.085). Distinct distribution patterns among the 0.1 BC, 0.5 BC, and control groups were similarly observed in the PCA score plot, although some overlap among groups remained evident (Figure 2b). Although some visual clustering was observed, PERMANOVA showed no significant differences among groups (*p* = 0.39).

Putatively annotated metabolites identified using the Bruker MetaboBASE Personal Library 2.0 (Bruker Daltonics GmbH, Bremen, Germany) were used to evaluate the biochemical shifts driving group separation in this dataset. The Random Forest variable importance analysis identified several metabolites that contributed strongly to discrimination among the 0.1 BC, 0.5 BC, and control groups (Figure 2c). Mean Decrease Accuracy showed that higher values indicate greater importance of a metabolite in classification performance. The compound 4-amino-2-hydroxybutyric acid was identified as the most critical variable for classification, followed closely by the fatty acid (9Z,12E)-15,16-epoxy-11-hydroxyoctadeca-9,12-dienoic acid, 2-hydroxyhexadecanoic acid, and L-Tyrosine, which were key primary metabolites. These compounds played major roles in distinguishing the treatment groups from the control.

All treatment groups demonstrated clear treatment-dependent metabolic variations. The prominent metabolites (e.g., L-tyrosine, biotin, 3-cyano-L-alanine, threonate, Gly-Gly, and butabarbital) were higher in the 0.1 BC group compared with the 0.5 BC and control groups. In contrast, the 4-amino-2-hydroxybutyric acid metabolite exhibited the highest abundance in the control samples. Additionally, L-histidine and the fatty acid metabolite were expressed at greater levels in the 0.5 BC treatment. The present results suggested dose-specific metabolic responses associated with BC fortification. However, confirmation with authentic standards is needed to verify these metabolite identities.

#### 3.2.2. Principal Component Analysis (PCA) from Positive Mode

**Figure 3:**
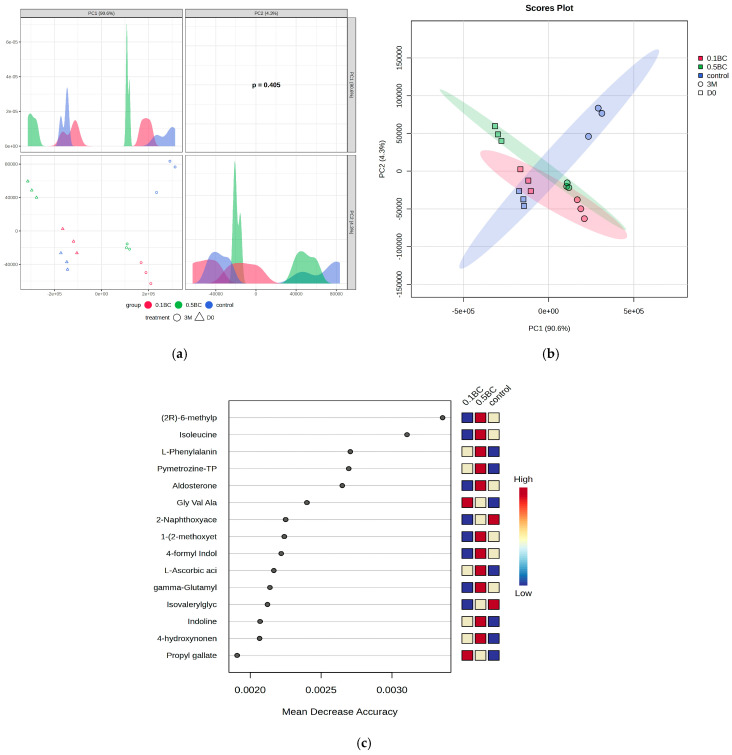
Principal components analysis (PCA) of Metabolite Profiles in the Snacks analyzed by GC-MS in Positive mode: (**a**) PCA, (**b**) PCA score plot, (**c**) Random Forest variable importance plot. The heatmap shows the relative abundance patterns across groups, with red indicating higher abundance and blue indicating lower abundance.

Principal component analysis (PCA) of metabolite composition revealed clear discrimination among the treatment groups (Figure 3a). The PCA score plot showed distinct clustering patterns among the 0.1 BC, 0.5 BC, and control groups, with slight overlap among the treatment groups (Figure 3b). Separation by storage period between day-0 and 3-month samples was also observed. Visual grouping was observed in the PCA; however, PERMANOVA analysis indicated that the overall differences in metabolomic profiles among groups were not statistically significant (*p* = 0.405).

Analysis of putatively annotated metabolites, identified by matching MS/MS spectra and retention times to the Bruker MetaboBASE Personal Library 2.0 (Bruker Daltonics GmbH, Bremen, Germany), revealed treatment-dependent metabolic alterations across the treatment groups. Based on Mean Decrease Accuracy (MDA) (Figure 3c), the compound annotated as (2R)-6-methylpiperidine-2-carboxylic acid exhibited the highest importance score, followed by isoleucine, L-phenylalanine, pymetrozine-TP, and aldosterone. These metabolites showed greater abundance in the 0.5 BC treatment (MDA > 0.0025). In contrast, metabolites such as Gly-Val-Ala and propyl gallate accumulated predominantly in the 0.1 BC group. In the control snack, 2-naphthoxyacetic acid and isovaleryglycine accumulated in abundance. However, confirmation with authentic standards is needed to verify these metabolite identities.

### 3.3. Top Discriminant Metabolites and Probiotic-Driven Matrix Changes During Storage

#### 3.3.1. Comparative Metabolite Profiles and Temporal Stability Shifts in Negative Mode

**Figure 4:**
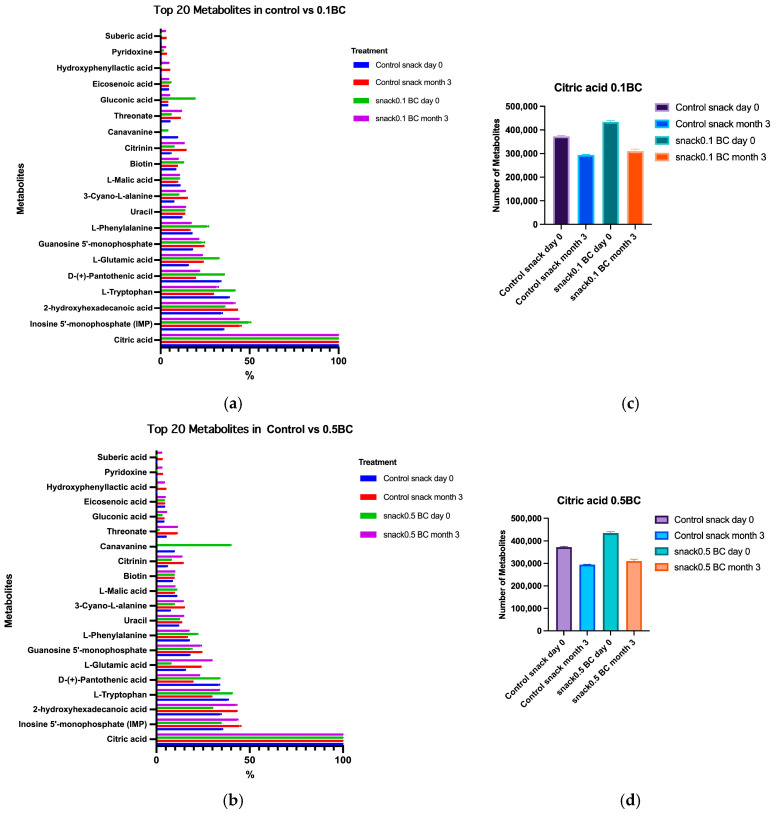
The Metabolite Profiles (negative mode) in the BC-Fortified Extruded Mung Bean Snacks, (**a**,**b**) The percentage of Metabolite Profiles normalized for each treatment compared with the control. (**c**,**d**) The number of the most abundant citric acid for each treatment compared with the control.

The abundances of the top 20 discriminative metabolites were monitored to determine the chemical profile of the functional snacks over a 3-month storage period. The baseline matrix of the control showed a significant difference in metabolite numbers at *p* < 0.01. Across all treatments, citric acid was identified as the predominant organic acid (Figure 4a–d) with an abundance of 100% (Figure 4a) and ranged from 294,284.7 to 434,196.0 metabolites (Table 2). The following abundances were Inosine 5′-monophosphate (IMP), 2-hydroxyhexadecanoic acid, L-tryptophan, D-(+)-Pantothenic acid, and L-glutamic acid. In the control snack, L-glutamic acid, 3-Cyano-L-alanine, Citrinin, and Threonate. The 0.5 BC formulation increased Threonate (19,071.3 metabolites) and 2-hydroxyhexadecanoic acid (15,489.3 metabolites), whereby several additional metabolites (e.g., Inosine 5′-monophosphate (IMP), L-glutamic acid, 3-Cyano-L-alanine were increased within 3 months. In contrast, all metabolites determined in the 0.1 BC group were decreased during the 3 months. Over a 3-month storage period, citric acid was reduced in all treatments.

#### 3.3.2. Comparative Metabolite Profiles and Temporal Stability Shifts in Positive Mode

**Figure 5:**
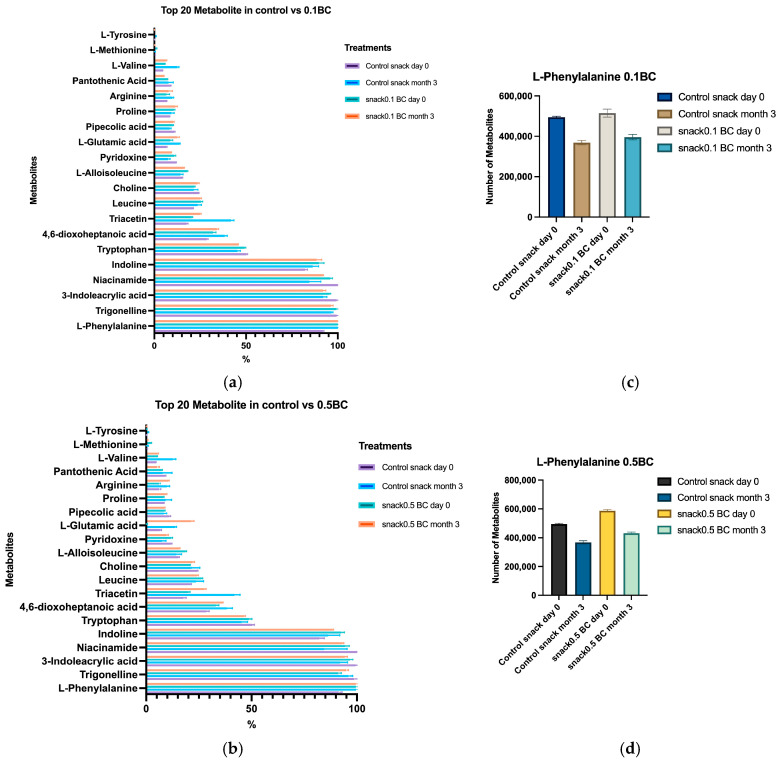
The Metabolite Profiles (positive mode) in the BC-Fortified Extruded Mung Bean Snacks, (**a**,**b**) The percentage of Metabolite Profiles normalized for each treatment compared with the control. (**c**,**d**) The number of the most abundant citric acid for each treatment compared with the control.

Both the 0.1 BC and 0.5 BC supplemented formulations at day 0 and month 3 showed a significant difference in metabolite numbers at *p* < 0.01 (Table 3). L-phenylalanine was identified as the predominant metabolite (Figure 5a–d), with 100% abundance, and the number of metabolites ranged from 368,630.7 to 587,224.7. L-phenylalanine was reduced in all treatments for 3 months (Figure 5c,d). The two doses exhibited distinct, dose-dependent effects during storage. In the control snack, Triacetin was markedly increased in 43,360 metabolites, 7-prenyl-theophylline, L-valine, L-glutamic acid, and ferulic acid. The 0.1 BC formulation induced immediate, significant increases in 7-prenyl-theophylline only (by 531 metabolites). The 0.5 BC formulation increased L-glutamic acid by about 68,930 metabolites within 3 months, and the following abundances were 7-prenyl-theophylline, 1-Decanamine, and L-isoleucine, while experiencing a significant reduction in free essential amino acids (L-valine, proline, and L-alloisoleucine).

### 3.4. Functional Annotation and Pathway Analysis of Differential Metabolites

#### 3.4.1. Altered Metabolic Pathways Identified in Negative Ionization Mode

**Figure 6:**
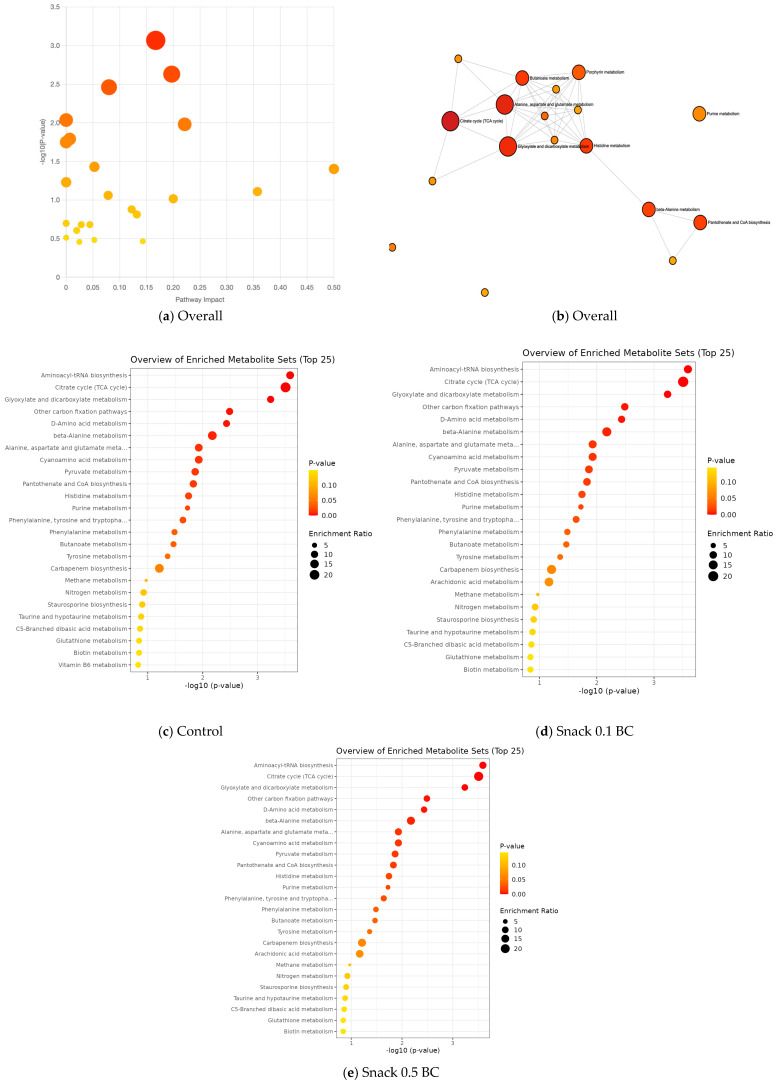
Identification of metabolic pathway and pathway enrichment analysis using MetaboAnalyst 6.0 based on the KEGG pathway library in electrospray negative ionization mode. (**a**) Metabolic pathway impact analysis of significantly altered metabolites; (**b**) Network topology: Nodes represent individual metabolic pathways, colored by their significance gradient (darker red indicates higher significance) and sized by their enrichment impact, and Connecting lines indicate shared biochemical intermediates or overlapping enzymatic networks. The top 25 enriched metabolite sets for (**c**) the Control, (**d**) Snack 0.1 BC, and (**e**) Snack 0.5 BC experimental groups.

Based on the KEGG library, pathway enrichment analysis showed several significant alternations in metabolic pathways (Figure 6a,b). Both analyses revealed that the Citrate cycle (TCA cycle) and Glyoxylate and dicarboxylate metabolism exhibited both high pathway impact values at 3 and 2.6 (−log 10 (*p*-value)) and strong significant enrichment in all treatments over 3 months of storage. Network topology analysis further demonstrated that alanine, aspartate, and glutamate metabolism, Histidine metabolism, and porphyrin metabolism showed a high impact and formed the network with the TCA cycle. Other pathways (e.g., purine metabolism, β-alanine metabolism, butanoate metabolism, and pantothenate and CoA biosynthesis) also contributed to the observed metabolic alterations with a lower impact. In all three treatments (Figure 6c–e), aminoacyl-tRNA biosynthesis and the TCA cycle were enriched within the top 25 sets as the most impacted pathways, suggesting that the core metabolic being stable with statistical significance (*p* < 0.05). Glyoxylate and dicarboxylate metabolism, other carbon fixation pathways, and D-amino acid metabolism maintained stable enrichment patterns across the groups. Lower-ranked pathways with lower significance profiles (*p* < 0.10) were glutathione and biotin metabolism, showing a minor drop in their overall ranking in the snack supplementation.

#### 3.4.2. Altered Metabolic Pathways Identified in Positive Ionization Mode

**Figure 7:**
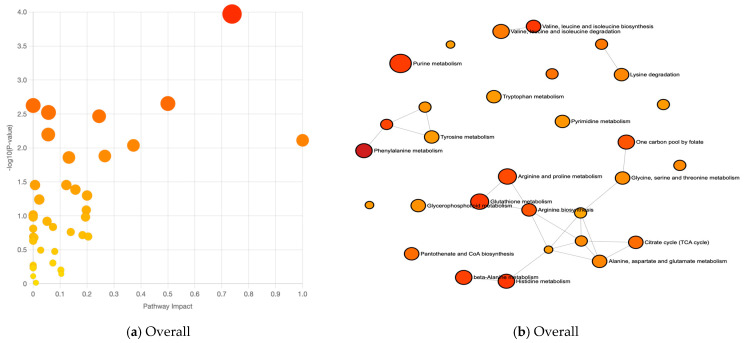

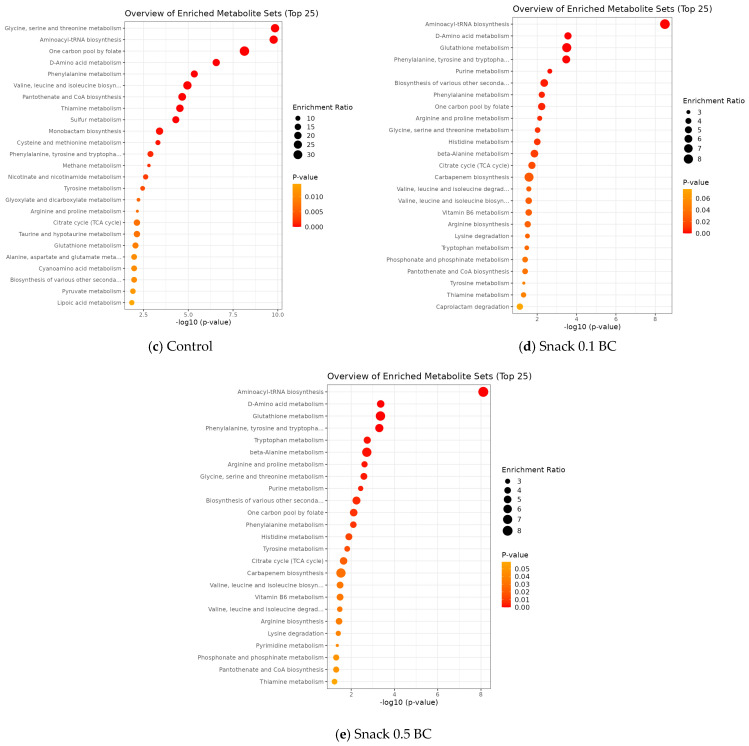
Identification of metabolic pathway and pathway enrichment analysis using MetaboAnalyst 6.0 based on the KEGG pathway library. (**a**) Metabolic pathway impact analysis of significantly altered metabolites identified in electrospray positive ionization mode. (**b**) Network topology: Nodes represent individual metabolic pathways, colored by their significance gradient (darker red indicates higher significance) and sized by their enrichment impact, and Connecting lines indicate shared biochemical intermediates or overlapping enzymatic networks. The top 25 enriched metabolite sets for (**c**) the Control, (**d**) Snack 0.1 BC, and (**e**) Snack 0.5 BC experimental groups.

Based on the pathway enrichment analysis in positive ionization mode, several significant alterations in amino acid-related metabolic pathways were observed (Figure 7a,b). Both analyses revealed that phenylalanine metabolism exhibited one of the highest levels of statistical significance. Network topology analysis further demonstrated that phenylalanine metabolism formed a metabolic network with the amino acid and was closely connected to the cluster with tyrosine metabolism.

Other pathways (e.g., arginine and proline metabolism, glycine, serine and threonine metabolism, alanine, aspartate and glutamate metabolism, and glutathione metabolism) also contributed to the observed metabolic alterations. In all treatment groups of 0.1 BC and 0.5 BC snacks (Figure 7c–e), Aminoacyl-tRNA biosynthesis was enriched as the most significantly impacted pathway with a major involvement of protein processes (*p* < 0.02). The control treatment showed strong enrichment in glycine, serine, and threonine metabolism and one-carbon pool by folate metabolism (*p* < 0.02). D-amino acid metabolism, glutathione metabolism, phenylalanine, tyrosine, and tryptophan biosynthesis were enriched pathways in all groups. Lower-enriched pathways differed between the treatment groups. Purine metabolism and lipid acid metabolism showed greater significance in the control, whereby pantothenate and CoA biosynthesis and thiamine metabolism showed reduced enrichment significance in the snack supplementation groups.

## 4. Discussion

The incorporation of probiotic spore (*B. coagulans*) into extruded snacks represents a significant advancement in functional food technology, transforming conventional snack products into functional snacks. The present study demonstrates that the extruded mung bean snacks serve as a delivery vehicle for *B. coagulans*. The present research provided metabolomic insights into discriminatory metabolite changes in amino acid metabolism, lipid oxidation, and flavor development in quality and storage stability. Similar to mung bean research by Sun et al. [25], reporting the analysis of the effects of hot processing (baking and cooking) on mung bean flavour, and the subsequent changes during storage, revealed that differential metabolites were involved in 145 metabolic pathways. Flavour compounds were primarily generated through glycolysis, the citric acid cycle, amino acid metabolism, and fatty acid degradation pathways.

### 4.1. Probiotic Survival in Fortified Extruded Mung Bean Snacks for 3 Months of Storage

The results of the study revealed that *B. coagulans* survival was maintained throughout the 3-month storage period, with viable counts above the recommended minimum level of 6–7 log CFU/g to gain a benefit from the probiotic consumption. In addition to plate counts, qPCR analysis detected *B. coagulans* DNA throughout the storage period. However, because qPCR quantifies bacterial DNA rather than viable cells, these results should be interpreted as evidence of DNA persistence and not as a direct measure of probiotic viability, survival, or functional delivery.

The discrepancy between culture-dependent counts and qPCR measurements may reflect differences in the analytical methods. While viable but non-culturable (VBNC) cells have been reported in bacteria under environmental stress [26], the present study did not directly assess the VBNC state. Therefore, the involvement of VBNC cells remains a possible explanation but cannot be confirmed based on the current experimental design. Additional approaches, such as viability PCR or flow cytometry, would be required to distinguish viable, dead, and VBNC cells.

The delivery of the *B. coagulans* probiotic remains effective in extruded mung bean snacks with *B. coagulans.* The persistence of *B. coagulans* observed during storage may be attributed, at least in part, to its spore-forming capacity. The *B. coagulans* persistence may be due to its spore-forming capacity over a three-month storage period. Its spores confer resistance against environmental stresses, including low water activity, oxidative stress, thermal processing, and prolonged storage conditions [26]. A previous study reported that *B. coagulans* thrived at extrusion barrel temperatures and that its spores can survive higher temperature exposures [27], allowing them to withstand the mechanical loads of the extrusion process.

The observed *B. coagulans* stability also suggests that the extrusion matrix provided a sufficient protective microenvironment by restricting oxygen diffusion and reducing structural degradation during storage. The additional starch-protein matrix of mung beans may provide a secondary protective barrier against physical degradation [26]. Low-moisture food matrices support probiotic persistence, as dehydrated probiotics enhance long-term viability because low water availability limits biochemical reactions, leading to improved storage stability [28]. In a similar experiment of quinoa extruded snacks with 0.3% *B. coagulans* GBI-30, Muñoz Pabon et al. [29] reported that the probiotic viability was above 10^7^ CFU/g during 120 days of storage at room temperature and had a survival rate of 70% under gastric conditions, preserving the functional potential of probiotics until it reaches the host’s gastrointestinal tract. In accordance with previous research [29], our products can ensure high probiotic viability throughout a 3-month storage period.

Differences between the low-dose (0.1 BC) and high-dose (0.5 BC) formulations suggested that BC formulation-dependent interactions significantly influenced microbial behavior. The higher stability of the 0.5%BC treatment may be due to an interaction between probiotic spores, matrix composition, and storage-associated metabolic changes, creating a protective microenvironment that favors bacterial survival kinetics. It can contribute to cell attachment and provide favorable niches, which may enhance probiotic persistence during storage [26].

### 4.2. Storage-Dependent Changes in Metabolite Profiles and Differential Abundance Patterns

From the LC-MS-based metabolomic analysis, BC supplementation induced selective modifications in metabolite abundance and metabolic pathway organization and contributed to dose-dependent redistribution of metabolites. Gradual shifts in several amino acids, organic acids, and nucleotide derivatives in the snack matrix were observed during storage. These substrates serve as stress-response mediators, changing metabolites within the snack matrix reflect adaptive microbial responses. *B. coagulans* fortification selectively modifies metabolite abundance patterns and metabolic pathway responses rather than causing large-scale restructuring of the entire metabolome [30]. This event was supported by PERMANOVA analysis, indicating no statistically significant differences in overall metabolomic composition among treatment groups. However, the clear cluster separation between day-0 and 3-month samples in both ionization modes indicated that storage duration remained the dominant factor driving metabolic variation. In the Random Forest analysis, several metabolites with high discriminatory power among treatments, suggesting that the amount of *B. coagulans* fortification affected specific biochemical processes in storage stability and metabolite turnover.

In the control snack, the 4-amino-2-hydroxybutyric acid metabolite was the most abundant. The 4-amino-2-hydroxybutyric acid was associated with amino acid metabolism [31]. Its abundance may be indicative of metabolic processes in the protein within the snack matrix. Accordingly, it can be used to determine microbial activity during product development. The identified flavor-associated nucleotides (IMP) and amino acids like L-tryptophan further suggested a shift in metabolites that characterizes the natural degradation of the snack over 3 months in the control group [32], although the appearance change was not detected. The unmodified mung bean matrix caused a lack of inherent microbial resilience, not only leading to a 42.2% reduction in *B. coagulans* levels but also exhibiting a significant surge in triacetin. From previous research, triacetin serves as a food additive approved by FAO/WHO JECFA [33]. The accumulation of triacetin was reduced in the *B. coagulans* fortification. It can be assumed that *B. coagulans* may prevent the lipid oxidation pathways that lead to sensorial deterioration [15,34].

Compared to the control group, the 0.1 BC treatment significantly altered the metabolic profiles, causing most major metabolites to rise. These findings highlight the strong impact of *B. coagulans* on amino acid metabolism and related pathways. Our results found that *B. coagulans* supported the retention of L-tyrosine and biotin, both of which are linked to antioxidant activity. L-tyrosine is an important aromatic amino acid that serves as a precursor for many phenolics and specialized metabolites, including tocopherols (vitamin E), which enhance plant-food functionality [35]. Biotin serves a different but vital purpose as a cofactor for carboxylases in fatty acid, carbohydrate, and energy metabolism. Therefore, its enrichment may reflect enhanced metabolic processes and preservation of key biochemical processes during storage. The 0.1 BC treatment may optimize the preservation or controlled accumulation of specific bioactive and antioxidant-associated compounds. Their enrichment may indicate that the low-dose matrix offers a favorable chemical environment during storage. Additionally, the 0.1 BC formulation induced a significant immediate increase in relative concentrations of organic acids and nucleotides, e.g., gluconic acid and IMP. Gluconic acid is a food-relevant organic acid derived from glucose oxidation and contributes to flavor, preservation, and product stability [36], whereas IMP is a key flavor-active nucleotide associated with umami taste and food quality [32].

The high dosage in the 0.5 BC treatment induced a systemic metabolic redistribution. Data from the negative-mode metabolomic profile confirmed that BC fortification selectively altered amino acid, organic acid, and lipid pathways. These changes may explain why the fortified snacks showed better preservation and distinct storage characteristics over time. The 0.5 BC snack accumulated L-glutamic acid and L-histidine while experiencing a significant reduction in free essential amino acids like L-valine and proline. Glutamate is a central metabolite in carbon and nitrogen metabolism, whereas valine and proline are closely associated with amino acid homeostasis and stress-responsive metabolic processes [37]. In the positive mode, enhancing essential amino acids such as isoleucine and L-phenylalanine in the 0.5 BC treatment indicated the promotion of selective retention or delayed degradation of protein-derived metabolites during storage [38]. Phenylalanine is closely linked to aromatic amino acid metabolism and secondary metabolite biosynthesis, while branched-chain amino acids such as isoleucine play important roles in energy homeostasis and stress adaptation, reflecting altered protein turnover kinetics or adsorption-mediated stabilization within the snack matrix [38]. Such shifts in amino acid profiles may reflect altered microbial metabolism and nitrogen utilization associated with the higher *B. coagulans* concentration. These changes may reflect differential microbial utilization, biosynthesis, or conversion of amino acids, leading to a distinct metabolic equilibrium different from that of the lower-dose formulation during storage [39]. The abundance of lipid-associated metabolites and L-histidine in the 0.5 BC treatment suggests that higher BC concentrations may alter membrane-related metabolic processes during storage. While these findings did not directly demonstrate membrane lipid turnover, they were consistent with treatment-dependent alterations in lipid metabolism and amino acid [39]. A similar study on the metabolome of the extruded snack identified changes in amino acids, organic acids, fatty acids, and phenolics [40]. They demonstrated that extrusion significantly altered metabolic profiles and may also affect nutrient-related compounds. Our results were in accordance with characterizing phytochemicals and metabolites in an extruded maize bean snack; extrusion modified the nutritional and metabolite composition of plant-based snacks [41].

Overall, BC supplementation induced selective modifications in metabolic restructuring within the snack matrix, particularly within pathways associated with central carbon metabolism, amino acid metabolism, organic acid and nucleotide metabolism, and storage-related biochemical transformations.

### 4.3. Linking Metabolite Alterations and KEGG Pathways to Functional Snack Quality and Storage Stability

In the non-fortified snack, our results showed that several metabolites associated with amino acid degradation, lipid metabolism, and organic acid turnover accumulated during storage, suggesting progressive biochemical changes within the plant-based matrix in a dose-dependent manner. These observations are consistent with previous studies showing that storage can alter protein, lipid, and carbohydrate-derived metabolites in extruded foods through oxidation and other degradation reactions [40]. Snacks fortified with *B. coagulans* showed distinct metabolite profiles characterized by the retention or enrichment of selected amino acids, organic acids, and nucleotide-related metabolites. In contrast, differences between fortified and non-fortified treatments indicated that the presence of probiotic spores may influence the biochemical processes of the food matrix during storage. Because the spore structure of *Bacillus species* provides excellent thermal protection during industrial extrusion, the core energy-yielding pathways remain metabolically stable post-processing [42]. The observed biochemical changes in pathway enrichment and topology analyses occurred through coordinated metabolic reorganization rather than isolated reactions [30]. Similar network-wide metabolic alterations in food metabolomic responses occur at key metabolic hubs, which reflect nutrient stability, biochemical reactions, and storage-associated transformations [43].

#### 4.3.1. Amino Acid Metabolism

Among the enriched metabolic pathways, the citrate cycle (TCA cycle) and glyoxylate and dicarboxylate metabolism were triggered by the *B. coagulans* supplementation. There were significantly impacted pathways, influencing the preservation and redistribution of organic acid intermediates associated with energy metabolism and oxidative balance. Citric acid remained highly stable throughout the 3-month storage period. The abundance of key TCA cycle components remained comparatively stable throughout storage. *B. coagulans* fortification was associated with the preservation of organic acid pools during storage. The enrichment of alanine, aspartate, and glutamate metabolism indicated coordinated changes in amino acid turnover and central carbon metabolism. These amino acids (L-glutamic acid) in the 0.5 BC group were closely connected to TCA cycle intermediates. This central metabolic hub may reflect the strong coupling between carbon metabolism and amino acid turnover within the fortified snack matrix. Alterations in this pathway were potentially related to shifts in carbon–nitrogen balance and energy-related metabolism during storage [44,45]. Together with the observed changes in organic acid-related pathways, it may further highlight the central role of this pathway in the biochemical response of the snack matrix. For instance, high concentrations of BC (0.5%) may promote a systemic redistribution of amino acids, such as the accumulation of L-glutamic acid, which serves as a critical bridge connecting nitrogen metabolism to the stable TCA cycle.

When considering the pathway in each treatment, the enrichment of aminoacyl-tRNA biosynthesis was similarly upregulated in both the 0.1 BC and 0.5 BC groups. Enrichment of aminoacyl-tRNA biosynthesis reflected coordinated changes in amino acid pools associated with translational processes [46]. A previous study showed that mung beans and other high-fiber/protein ingredients restricted radial expansion and increased bulk density in extruded pulse snacks, creating a compacted matrix that alters oxygen diffusion [47]. The system responds by downregulating standard amino acid degradation, shifting resources toward specific protein synthesis and structural maintenance instead of routine nutrient turnover [48]. Furthermore, downregulating peripheral vitamin cofactor pathways (such as pantothenate and thiamine metabolism) [49], it may suggest that this functional reprogramming successfully links a shelf-stable physical extrudate matrix with optimized, long-term probiotic survival.

#### 4.3.2. Aromatic Amino Acid Metabolism

Phenylalanine metabolism is one of the most significantly enriched metabolic pathways. The pathway was closely associated with tyrosine metabolism, reflecting the well-established biochemical relationship between these aromatic amino acid pathways. Several amino acid-related pathways, including tyrosine metabolism, tryptophan metabolism, arginine and proline metabolism, glycine, serine, and threonine metabolism, and alanine, aspartate, and glutamate metabolism, were also significantly represented, suggesting extensive remodeling of amino acid metabolism as a key biochemical feature underlying the observed metabolic alterations.

Changes in phenylalanine metabolism may reflect alterations in aromatic amino acid turnover. Since phenylalanine serves as a precursor to tyrosine, it can influence metabolic transformations and the generation of aromatic compounds [50]. These aromatic amino acid pathways play important roles in the formation of bioactive compounds, antioxidant activity, and flavor-related metabolites. Previous research reported that aromatic amino acids contribute to Maillard reactions and volatile compound formation [51], these changes may affect food flavor and sensory quality. In addition, the present study further suggests the appearance of the D-amino acid, glutathione, and aromatic amino acid (phenylalanine, tyrosine, and tryptophan) biosynthetic processes in the snack treatments. The upregulation of glutathione metabolism is particularly critical from a shelf-life perspective. Glutathione functions as a dominant endogenous antioxidant; its metabolic elevation implies a fortified cellular defense network capable of mitigating the oxidative stresses that accumulate over extended storage periods [52]. To maintain probiotic functionality over a standard ambient shelf life of up to 180 days, *Bacillus* counts must remain above the established therapeutic threshold (≥7–8 log CFU/g) in accordance with the viable cells in the snack with 0.5 BC formulation. Glutathione and aromatic amino acid pathways may contribute to a biochemical environment potentially favorable for probiotic stability [42,53].

#### 4.3.3. Purine Metabolism

Additional downstream alterations of porphyrin, purine, β-alanine, and pantothenate/CoA metabolism suggest that BC supplementation influenced cofactor biosynthesis, nucleotide metabolism, and redox-related processes. Purine metabolism also emerged as a highly significant pathway hub, indicating substantial changes in nucleotide-related metabolism. Alterations in this pathway are consistent with the observed variation in nucleotide-derived compounds, including inosine monophosphate and guanosine-related metabolites [13]. Because purine metabolites contribute to flavor development and are sensitive to degradation during storage, these changes may reflect differences in nucleotide turnover and preservation between treatments. Collectively, the results suggested that BC supplementation influenced both redox-associated metabolism and nucleotide stability, contributing to the distinct metabolomic profiles observed during storage [13]. Changes in guanosine monophosphate further support an effect on biochemical stability during storage.

#### 4.3.4. Lipid Oxidation

A shift in bioactive and antioxidant-associated compounds, such as L-tyrosine and biotin, was observed in the 0.1 BC fortification level. This finding may indicate that *B. coagulans* in the snack delayed this degradation pathway. The pathway interaction network revealed close links among arginine biosynthesis, arginine and proline metabolism, and glutathione metabolism [34]. These pathways are involved in nitrogen utilization and cellular redox balance, suggesting that BC supplementation influenced metabolic processes linked to antioxidant capacity during storage. Organic acids such as gluconic acid and threonate increased over the storage period [16]. Their accumulation may have contributed to reduced oxidative changes and supported metabolite preservation. Changes in glutathione metabolism are particularly important in maintaining redox balance and protecting cellular components from oxidative damage. This pathway may have contributed to supporting the biochemical processes observed in BC-fortified samples [54].

In extruded foods, the formation of amylose–lipid complexes can reduce oxygen diffusion and improve the stability of lipid-containing matrices [52]. In addition, alterations in glutathione and biotin metabolism were observed among the snack groups, suggesting a redistribution of metabolites involved in antioxidant defense and cofactor-dependent metabolic processes. These metabolic changes may have contributed to the reduced formation of oxidation-related compounds during storage. Similar metabolomic responses have been reported in refrigerated beef, where microbial activity significantly altered metabolic pathways, particularly histidine metabolism, and led to the accumulation of spoilage-associated biomarkers such as inosine [34].

Minor degradation of valine, leucine, and isoleucine, which was also significantly affected by BC supplementation. These amino acids play important roles in protein turnover, nitrogen metabolism, and energy production [55]. Similar alterations in amino acid profiles have been reported during food processing and storage, where changes in amino acid metabolism are closely associated with oxidative reactions, protein degradation, and quality deterioration [55]. The reduction of certain valine, leucine, and isoleucine under high BC conditions may be associated with adsorption-mediated retention within the matrix or increased conversion into downstream metabolites involved in energy-generating pathways [42,43,44].

### 4.4. Limitation

The metabolomic analysis was based on a limited number of biological replicates (*n* = 2); therefore, the findings should be considered exploratory and interpreted with caution. In addition, because metabolomics was performed on the whole snack matrix, the relative contributions of microbial metabolism and storage-related chemical reactions could not be distinguished. Metabolite annotations were based on matching MS/MS spectra and retention times to the Bruker MetaboBASE Personal Library 2.0 (Bruker Daltonics GmbH, Bremen, Germany) and thus represent putative identifications that require confirmation using authentic reference standards. Likewise, pathway enrichment analysis should be regarded as exploratory because it depends on metabolite coverage and KEGG annotations. The Random Forest analysis, performed in MetaboAnalyst 6.0 using the Mean Decrease Accuracy metric, was applied as an exploratory feature-ranking method rather than a validated predictive classification model. Therefore, further studies with larger sample sizes and complementary analytical approaches are needed to validate the identified metabolites, metabolic pathways, and discriminatory features and to clarify the underlying biological mechanisms.

## 5. Conclusions

This study demonstrated that culturable *B. coagulans* was maintained in fortified extruded mung bean snacks throughout 3 months of storage. The 0.5%BC formulation was the most stable in the snack, with viable counts of 7.10 log CFU/g. The distinct metabolic profile of each treatment showed dose-dependent metabolic variations. The qPCR analysis indicated a higher abundance of *B. coagulans* DNA in the 0.5%BC treatment, with a 26.6% increase in the DNA threshold compared with the initial measurement. In contrast, a 42.2% reduction of the DNA threshold in the control group was detected. From the changes in the metabolomic data, the 0.1%BC snack accumulated L-tyrosine, biotin, and threonate, whereas the 0.5%BC snack upregulated L-glutamic acid and L-histidine while depleting free essential amino acids (L-valine and proline). The TCA cycle and aminoacyl-tRNA biosynthesis were identified as the primary pathways across all treatments, with citric acid and L-phenylalanine abundance. Within 3 months, metabolomic analysis suggested changes associated with amino acid metabolism, lipid oxidation, and flavor development; however, these findings should be considered exploratory. *B. coagulans* fortification was associated with lower triacetin abundance, which may indicate changes related to lipid oxidation during storage. Pathway analysis suggested the involvement of glutathione metabolism, D-amino acid metabolism, and aromatic amino acid metabolism; however, further validation is required. Together, these findings provide preliminary insights into metabolite changes associated with probiotic spore fortification and may assist future studies aimed at identifying potential markers of product quality and storage stability. Future studies combining other omics approaches and longer storage studies, including sensory quality and texture properties, would provide a more comprehensive evaluation of changes in developed functional attributes of plant-based extruded products for commercial use.

## Figures and Tables

**Table 1:** The primer lists for qPCR.

Primer	Sequences
Allbac-F	AAACTCAAAKGAATTGACGG
Allbac-R	CTCACRRCACGAGCTGAC
BC-F	TCGTGTCGTGAGATGTTGGG
BC-R	ACCTTCCTCCGGTTTGTCAC

**Table 2:** The Detected Metabolite Profiles (negative mode) in the BC-Fortified Extruded Mung Bean Snacks for 3 months of storage.

Metabolite	Control D0	Control M3	0.1 BC D0	0.1 BC M3	0.5 BC D0	0.5 BC M3	*p*-Value
Citric acid	372,279.3 ± 4319.5	294,284.7 ± 2198.2	434,196.0 ± 6496.1	310,653.3 ± 8149.7	368,662.0 ± 6144.7	314,196.7 ± 177,642.1	<0.01
Inosine 5′-monophosphate (IMP)	138,441.3 ± 3107.0	130,496.0 ± 4771.3	222,929.3 ± 9034.8	136,641.3 ± 1621.1	135,726.7 ± 378.8	136,625.3 ± 79,640.7	<0.01
2-Hydroxyhexadecanoic acid	134,583.3 ± 4700.2	126,523.3 ± 1234.8	165,812.0 ± 2965.1	127,766.7 ± 4712.3	118,972.7 ± 1570.2	134,462.0 ± 76,848.2	<0.01
L-Tryptophan	149,547.3 ± 2993.9	86,680.0 ± 1522.3	189,643.3 ± 2571.6	98,001.3 ± 6274.2	156,412.0 ± 1298.2	106,744.7 ± 650,599.1	<0.01
D-(+)-Pantothenic acid	132,038.0 ± 4053.6	57,649.3 ± 211.7	164,870.0 ± 2120.8	67,204.0 ± 2605.1	132,084.0 ± 2606.7	73,762.7 ± 41,295.5	<0.01
L-Glutamic acid	67,234.0 ± 2212.8	69,730.7 ± 1330.3	152,327.3 ± 2283.5	72,571.3 ± 1740.9	39,465.3 ± 1435.4	94,525.3 ± 53,573.5	<0.01
Guanosine 5′-monophosphate	75,686.7 ± 2418.2	70,900.0 ± 1605.7	112,707.0 ± 11,943.3	66,707.3 ± 1234.6	77,422.0 ± 5428.6.	75,450.0 ± 41,275.8	<0.01
L-Phenylalanine	75,075.0 ± 1596.9	47,384.7 ± 2558.8	125,550.0 ± 6379.0	52,888.7 ± 2171.7	89,412.0 ± 4058.2	55,164.0 ± 30,148.9	<0.01
Uracil	54,570.0 ± 1641.0	39,462.7 ± 784.4	72,941.3 ± 944.2	43,700.7 ± 1165.0	56,113.3 ± 778.3	46,282.7 ± 26,524.8	<0.01
3-Cyano-L-alanine	38,450.7 ± 1375.1	43,231.3 ± 1709.0	57,578.7 ± 2891.9	43,634.0 ± 775.7	45,132.0 ± 2658.5	45,776.7 ± 25,942.5	<0.01
L-Malic acid	51,160.0 ± 1135.2	27,150.0 ± 2103.1	59,747.3 ± 2022.4	33,684.0 ± 453.2	50,523.3 ± 1467.4	31,720.0 ± 17,929.9	<0.01
Biotin	42,749.3 ± 820.4	27,402.7 ± 684.2	69,433.3 ± 1695.9	31,266.7 ± 667.1	46,038.7 ± 803.7	31,893.3 ± 18,248.4	<0.01
Citrinin	31,984.0 ± 2786.8	41,412.7 ± 1368.9	46,717.3 ± 2872.6	41,454.7 ± 930.0	40,052.0 ± 1424.2	43,574.0 ± 24,679.9	<0.01
Canavanine	46,132.7 ± 1248.3	169.3 ± 62	32,570.7 ± 1811.2	1173.3 ± 120.7	152,971.3 ± 3200	1939.3 ± 676.9	<0.01
Threonate	30,811.3 ± 1043.9	31,831.3 ± 1545.4	40,406.0 ± 1870.9	36,774.0 ± 1018.4	17,430.0 ± 476.8	36,501.3 ± 20,925.7	<0.01
Gluconic acid	26,313.3 ± 870.3	11,800.0 ± 285.7	96,594.7 ± 1264.3	16,118.0 ± 1068.7	22,762.7 ± 553.0	18,566.7 ± 10,591.2	<0.01

Values are presented as Mean ± SD (*n* = 3 replicates per treatment).

**Table 3:** The Detected Metabolite Profiles (positive mode) in the BC-Fortified Extruded Mung Bean Snacks for 3 months of storage.

Metabolite	Control D0	Control M3	0.1 BC D0	0.1 BC M3	0.5 BC D0	0.5 BC M3	*p*-Value
L-Phenylalanine	494,488.7 ± 5223.9	368,630.7 ± 11,346.0	514,563.3 ± 19,937.7	395,998.7 ± 13,402.9	587,224.7 ± 7468.6	431,668.7 ± 8214.3	<0.01
Trigonelline	531,516.0 ± 8811.4	356,956.0 ± 4462.1	510,410.0 ± 16,174.0	382,148.0 ± 7739.9	542,863.3 ± 4032.9	413,036.7 ± 2206.8	<0.01
3-Indoleacrylic acid	531,383.3 ± 6519.8	341,048.7 ± 12,147.3	493,640.7 ± 4302.0	366,126.7 ± 9463.2	569,393.3 ± 7191.9	408,281.3 ± 5027.6	<0.01
Niacinamide	534,978.0 ± 4210.6	315,069.3 ± 37,325.9	495,310.7 ± 9128.3	365,413.3 ± 2872.1	557,305.3 ± 9999.6	403,538.3 ± 2736.2	<0.01
Indoline	445,988.0 ± 11,617.4	321,470.0 ± 18,981.6	465,429.3 ± 22,608.2	352,804.7 ± 18,449.3	546,516.0 ± 8070.9	384,269.3 ± 2216.8	<0.01
Tryptophan	286,752.7 ± 5729.2	179,722.7 ± 9527.7	271,994.7 ± 4907.5	194,547.3 ± 720.8	308,856.0 ± 7307.4	213,804.0 ± 3028.8	<0.01
4,6-Dioxoheptanoic acid	178,983.3 ± 6413.9	155,576.7 ± 8953.9	189,634.0 ± 13,065.9	151,478.7 ± 5732.9	223,742.7 ± 7525.0	172,278.3 ± 1881.8	<0.01
Triacetin	124,379.3 ± 6825.38	167,739.3 ± 9359.5	134,050.0 ± 4293.4	117,314.7 ± 3728.0	153,178.7 ± 3704.8	137,679.0 ± 3884.8	<0.01
Leucine	141,787.3 ± 1853.1	104,952.7 ± 12,352.9	158,480.7 ± 6853.1	117,736.0 ± 4504.0	186,492.0 ± 3035.4	123,933.0 ± 2737.3	<0.01
Choline	156,808.7 ± 2273.1	97,677.3 ± 13,414.6	141,028.0 ± 3202.3	112,246.7 ± 5552.3	156,848.0 ± 557.7	115,198.7 ± 3837.8	<0.01
L-Alloisoleucine	112,287.3 ± 2771.2	72,547.3 ± 8641.4	121,769.3 ± 3456.1	84,330.7 ± 2541.0	145,217.3 ± 2488.3	89,588.3 ± 1615.2	<0.01
Pyridoxine	95,382.7 ± 2259.0	49,182.0 ± 6686.8	88,621.3 ± 6200.1	58,621.3 ± 1985.5	108,084.0 ± 3543.6	65,234.0 ± 3633.8	<0.01
L-Glutamic acid	69,176.0 ± 3643.1	70,633.3 ± 2352.5	78,360.7 ± 10,745.4	71,614.7 ± 6806.5	43,923.3 ± 3207.8	112,853.7 ± 5290.0	<0.01
Pipecolic acid	90,982.0 ± 3495.8	52,241.3 ± 4583.9	84,651.3 ± 3145.6	63,046.7 ± 3406.9	90,494.0 ± 2000.9	61,116.0 ± 1317.1	<0.01
Proline	78,354.0 ± 869.6	55,310.0 ± 9481.0	87,240.0 ± 5134.2	67,414.0 ± 6933.0	88,186.0 ± 1520.8	64,033.7 ± 2035.9	<0.01
Arginine	70,166.7 ± 1004.4	57,178.0 ± 4619.0	68,056.0 ± 14,199.9	53,696.7 ± 1375.7	79,662.7 ± 225.0	68,348.0 ± 2048.7	0.03
Pantothenic Acid	81,074.7 ± 2156.2	50,020.7 ± 15,538.7	70,414.7 ± 1848.6	43,867.3 ± 1143.9	83,953.3 ± 1379.4	49,211.7 ± 2411.2	<0.01
L-Valine	59,664.0 ± 278.6	66,750.7 ± 5153.3	64,170.7 ± 859.2	48,068.0 ± 3337.9	71,698.7 ± 1317.7	47,469.3 ± 2304.7	<0.01
Pyroglutamic acid	62,426.7 ± 2145.7	40,900.7 ± 5264.5	63,882.7 ± 2187.1	46,101.3 ± 1208.4	75,091.3 ± 779.3	47,989.3 ± 1332.1	<0.01
L-Methionine	39,384.7 ± 394.7	23,473.3 ± 3462.7	42,099.3 ± 1624.8	24,902.0 ± 1864.2	55,640.0 ± 2018.0	28,518.3 ± 1021.8	<0.01
7-Prenyl-theophylline	33,728.7 ± 1947.4	49,344.0 ± 12,243.1	33,814.0 ± 178.5	34,345.3 ± 465.7	37,389.3 ± 2185.0	40,374.3 ± 2340.9	0.02
1-Decanamine	31,201.3 ± 768.2	30,182.7 ± 1436.7	32,540.0 ± 2344.3	31,964.7 ± 1229.1	32,788.0 ± 478.4	35,629.0 ± 3120.1	0.04
Citric acid	4944.0 ± 71.1	4509.3 ± 650.2	4774.7 ± 34.4	4002.7 ± 405.8	4868.0 ± 78	4185.0 ± 92.0	0.02
Ferulic acid	4230.7 ± 329.1	4326.0 ± 178.5	4371.3 ± 84.2	3980.0 ± 323.5	5526.7 ± 963.6	4392.3 ± 107.2	0.02
L-Isoleucine	3290.7 ± 113.0	2603.3 ± 986.2	4097.3 ± 295.6	3824.0 ± 461.1	3930.7 ± 113.7	4279.3 ± 928.4	0.04

Values are presented as Mean ± SD (*n* = 3 replicates per treatment).

## Data Availability

The original contributions presented in this study are included in the article. Further inquiries can be directed to the corresponding authors.

## References

[1] Sanap S., Peterson L. (2026). Global Mung Bean Market Size, Industry Trends & Forecast 2026–2034.

[2] Hou D., Yousaf L., Xue Y., Hu J., Wu J., Hu X., Feng N. (2019). Mung bean (*Vigna radiata*): Bioactive compounds, health benefits, and applications. Food Sci. Nutr..

[3] Singh S., Gamlath S., Wakeling L. (2007). Nutritional aspects of food extrusion: A review. Int. J. Food Sci. Technol..

[4] Brennan C., Brennan M., Derbyshire E., Tiwari B. (2013). Effects of extrusion on the polyphenols, vitamins and antioxidant activity of foods. Trends Food Sci. Technol..

[5] Hill C., Guarner F., Reid G., Gibson G.R., Merenstein D.J., Pot B., Morelli L., Canani R.B., Flint H.J., Salminen S. (2014). Expert consensus document: The International Scientific Association for Probiotics and Prebiotics consensus statement on the scope and appropriate use of the term probiotic. Nat. Rev. Gastroenterol. Hepatol..

[6] Sanders M.E., Merenstein D., Reid G., Gibson G.R., Rastall R.A. (2019). Probiotics and prebiotics in intestinal health and disease: From biology to the clinic. Nat. Rev. Gastroenterol. Hepatol..

[7] Elshaghabee F.M.F., Rokana N., Gulhane R., Sharma C., Panwar H. (2017). Bacillus as potential probiotics: Status, concerns and future perspectives. Front. Microbiol..

[8] Payne J., Bellmer D., Jadeja R., Muriana P. (2024). The Potential of *Bacillus* Species as Probiotics in the Food Industry: A Review. Foods.

[9] Konuray G., Erginkaya Z. (2018). Potential Use of *Bacillus coagulans* in the Food Industry. Foods.

[10] Majeed M., Majeed S., Nagabhushanam K., Natarajan S., Sivakumar A., Ali F. (2016). Evaluation of the stability of *Bacillus coagulans* MTCC 5856 during processing and storage of functional foods. Int. J. Food Sci. Technol..

[11] Mani-López E., Ramírez-Corona N., López-Malo A. (2023). Advances in probiotic incorporation into cereal-based baked foods: Strategies, viability, and effects—A review. Appl. Food Res..

[12] Marco M.L., Heeney D., Binda S., Cifelli C.J., Cotter P.D., Foligné B., Gänzle M., Kort R., Pasin G., Pihlanto A. (2017). Health benefits of fermented foods: Microbiota and beyond. Curr. Opin. Biotechnol..

[13] Putri S.P., Ikram M.M.M., Sato A., Dahlan H.A., Rahmawati D., Ohto Y., Fukusaki E. (2022). Application of Gas Chromatography–Mass Spectrometry-Based Metabolomics in Food Science and Technology. J. Biosci. Bioeng..

[14] Zhang J., Sun M., Elmaidomy A.H., Youssif K.A., Zaki A.M.M., Kamal H.H., Sayed A.M., Abdelmohsen U.R. (2023). Emerging Trends and Applications of Metabolomics in Food Science and Nutrition. Food Funct..

[15] Fang J., Feng L., Lu H., Zhu J. (2022). Metabolomics Reveals Spoilage Characteristics and Interaction of Pseudomonas lundensis and Brochothrix thermosphacta in Refrigerated Beef. Food Res. Int..

[16] He K., Zhao M., Tong X., Feng Y. (2026). Reveal the flavor quality changes of soy sauce during shelf life through metabolomics. Food Chem..

[17] Zhang X., Zheng Y., Liu Z., Su M., Wu Z., Zhang H., Zhang C., Xu X. (2024). Insights into characteristic metabolites and potential bioactive peptide profiles of fresh cheese fermented with three novel probiotics based on metabolomics and peptidomics. Food Chem..

[18] Peng S., Guo C., Wu S., Cui H., Suo H., Duan Z. (2022). Bioactivity and metabolomics changes of plant-based drink fermented by *Bacillus coagulans* VHProbi C08. LWT.

[19] Boonyasirikul P., Charunuch C., Pongpipatpong M. (1996). Production of Mung Bean Snack by Using Twin-Screw Extruder. Food.

[20] Charunuch C., Puntaburt K., Peagpinit W., Veskijkul A., Pan-Utai W., Lowithun N. (2015). Mung Bean Snack Product Produced Using an Extruder. Thai Petty Patent.

[21] Corn Refiners Association (2007). Microbiological Methods XI-A-1: Mesophilic Aerobic Spore-Formers Pour Plate Method.

[22] U.S. Food and Drug Administration (2026). BAM Chapter 3: Aerobic Plate Count. Bacteriological Analytical Manual.

[23] U.S. Food and Drug Administration (2025). BAM R11: Butterfield’s Phosphate-Buffered Dilution Water. Bacteriological Analytical Manual.

[24] United States Pharmacopeia (2023). *Bacillus* *coagulans*. USP–NF Dietary Supplement Monographs.

[25] Sun J., Li Y., Cheng X., Zhang H., Yu J., Zhang L., Qiu Y., Diao J., Wang C. (2025). Metabolomic analysis of flavour development in mung bean foods: Impact of thermal processing and storage on precursor and volatile compounds. Foods.

[26] Poshadri A., Deshpande H.W., Khodke U.M., Katke S.D. (2022). *Bacillus coagulans* and its Spore as Potential Probiotics in the Production of Novel Shelf-Stable Foods. Curr. Res. Nutr. Food Sci..

[27] Acuff H.L., Aldrich C.G. (2022). Effects of Extrusion Specific Mechanical Energy and Dryer Conditions on the Survival of *Bacillus coagulans* GBI-30, 6086 for Commercial Pet Food Applications. Anim. Feed Sci. Technol..

[28] Marcial-Coba M.S., Knøchel S., Nielsen D.S. (2019). Low-moisture food matrices as probiotic carriers. FEMS Microbiol. Lett..

[29] Muñoz Pabon K.S., Hoyos Concha J.L., Solanilla Duque J.F. (2022). Quinoa extruded snacks with probiotics: Physicochemical and sensory properties. Front. Sustain. Food Syst..

[30] Zhu H., Wang H., Wang L., Zheng Z. (2025). Integrated Metabolomic and Transcriptomic Analyses Reveal Molecular Mechanisms of the Acid Stress Response in *Bacillus coagulans*. Food Biosci..

[31] Yukita T., Nishida H., Eguchi T., Kakinuma K. (2003). Biosynthesis of (2R)-4-Amino-2-hydroxybutyric Acid, Unique and Biologically Significant Substituent in Butirosins. J. Antibiot..

[32] Park M.K., Choi Y.S. (2025). Effective strategies for understanding meat flavor: A review. Food Sci. Anim. Resour..

[33] FAO Combined Compendium of Food Additive Specifications: Triacetin (Glyceryl Triacetate; INS 1518); Joint FAO/WHO Expert Committee on Food Additives (JECFA).

[34] Zhou Y., Zeng Z., Xu Y., Ying J., Wang B., Majeed M., Majeed S., Pande A., Li W. (2020). Application of *Bacillus coagulans* in Animal Husbandry and Its Underlying Mechanisms. Animals.

[35] Xu J.J., Fang X., Li C.Y., Yang L., Chen X.Y. (2019). General and Specialized Tyrosine Metabolism Pathways in Plants. Abiotech.

[36] Sirithanakorn C., Cronan J.E. (2021). Biotin, a Universal and Essential Cofactor: Synthesis, Ligation and Regulation. FEMS Microbiol. Rev..

[37] Shi Y., Pu D., Zhou X., Zhang Y. (2022). Recent Progress in the Study of Taste Characteristics and the Nutrition and Health Properties of Organic Acids in Foods. Foods.

[38] Ramachandran S., Fontanille P., Pandey A., Larroche C. (2006). Gluconic Acid: Properties, Applications and Microbial Production. Food Technol. Biotechnol..

[39] Marco M.L., Sanders M.E., Gänzle M., Arrieta M.C., Cotter P.D., De Vuyst L., Hill C., Holzapfel W., Lebeer S., Merenstein D. (2021). The International Scientific Association for Probiotics and Prebiotics (ISAPP) Consensus Statement on Fermented Foods. Nat. Rev. Gastroenterol. Hepatol..

[40] Song J., Tang Y. (2023). Effect of Extrusion Temperature on Characteristic Amino Acids, Fatty Acids, Organic Acids, and Phenolics of White Quinoa Based on Metabolomics. Food Res. Isnt..

[41] Félix-Medina J.V., Gutiérrez-Dorado R., López-Valenzuela J.A., López-Ángulo G., Quintero-Soto M.F., Perales-Sánchez J.X.K., Montes-Ávila J. (2021). Nutritional, Antioxidant and Phytochemical Characterization of Healthy Ready-to-Eat Expanded Snack Produced from Maize/Common Bean Mixture by Extrusion. LWT.

[42] Bento G.T., Santos A.Y.S., Rodrigues S., Fonteles T.V. (2026). Comparative Stability of Heyndrickxia coagulans Spores in Oat and Rice-Bean Matrices: Impact of Processing, Storage, and Simulated Digestion. Processes.

[43] Pang Z., Chong J., Zhou G., Anderson de Lima Morais D., Chang L., Barrette M., Gauthier C., Jacques P., Li S., Xia J. (2021). MetaboAnalyst 5.0: Narrowing the gap between raw spectra and functional insights. Nucleic Acids Res..

[44] Huergo L.F., Dixon R. (2015). The Emergence of 2-Oxoglutarate as a Master Regulator Metabolite. Microbiol. Mol. Biol. Rev..

[45] Wishart D.S. (2019). Metabolomics for Investigating Physiological and Pathophysiological Processes. Physiol. Rev..

[46] Ibba M., Söll D. (2000). Aminoacyl-tRNA Synthesis. Annu. Rev. Biochem..

[47] Aussanasuwannakul A., Kantrong H. (2026). Texture Phenotypes of Fiber-Enriched Extruded Snacks Revealed by Mechanical–Acoustic Analysis, Tribology, and Sensory Mapping. Foods.

[48] Njenga R., Boele J., Öztürk Y., Koch H.G. (2023). Coping with Stress: How Bacteria Fine-Tune Protein Synthesis and Protein Transport. J. Biol. Chem..

[49] Goyer A. (2010). Thiamine in Plants: Aspects of Its Metabolism and Functions. Phytochemistry.

[50] Hidalgo F.J., Zamora R. (2016). Amino Acid Degradations Produced by Lipid Oxidation Products. Crit. Rev. Food Sci. Nutr..

[51] Shahidi F., Yeo J. (2018). Bioactivities of Phenolics by Focusing on Suppression of Chronic Diseases: A Review. Int. J. Mol. Sci..

[52] Wang N., Wang X., Miao J., Dai J., Dai Y., Wang W., Hou H., You Z. (2025). Improved extrusion cooking technique (IECT) induces the formation of starch-lauric acid complexes: Evaluations of structure and properties. J. Future Foods..

[53] Alsanie S.A. (2026). Probiotic-fortified functional foods: Integrating nutrient delivery and gut health benefits. Front. Nutr..

[54] Dashdorj D., Amna T., Hwang I. (2015). Influence of Specific Taste-Active Components on Meat Flavor as Affected by Intrinsic and Extrinsic Factors: An Overview. Eur. Food Res. Technol..

[55] Cevallos-Casals B.A., Cisneros-Zevallos L. (2010). Impact of germination on phenolic content and antioxidant activity of 13 edible seed species. Food Chem..

